# Evidence of SARS-CoV-2 convergent evolution in immunosuppressed patients treated with antiviral therapies

**DOI:** 10.1186/s12985-024-02378-y

**Published:** 2024-05-07

**Authors:** Shuchen Feng, Gail E. Reid, Nina M. Clark, Amanda Harrington, Susan L. Uprichard, Susan C. Baker

**Affiliations:** 1 Department of Microbiology and Immunology, Stritch School of Medicine, Loyola University Chicago, Maywood, IL 60153 USA; 2 Department of Medicine, Stritch School of Medicine, Loyola University Chicago, Maywood, IL 60153 USA; 3 Infectious Disease and Immunology Research Institute, Stritch School of Medicine, Loyola University Chicago, Maywood, IL 60153 USA; 4 Department of Pathology and Laboratory Medicine, Stritch School of Medicine, Loyola University Chicago, Maywood, IL 60153 USA

**Keywords:** SARS-CoV-2 evolution, Convergent evolution, COVID-19, Immunosuppressed patients, iSNVs, Monoclonal antibody treatment, Antiviral treatment

## Abstract

**Background:**

The factors contributing to the accelerated convergent evolution of severe acute respiratory syndrome coronavirus 2 (SARS-CoV-2) are not fully understood. Unraveling the contribution of viral replication in immunocompromised patients is important for the early detection of novel mutations and developing approaches to limit COVID-19.

**Methods:**

We deep sequenced SARS-CoV-2 RNA from 192 patients (64% hospitalized, 39% immunosuppressed) and compared the viral genetic diversity within the patient groups of different immunity and hospitalization status. Serial sampling of 14 patients was evaluated for viral evolution in response to antiviral treatments.

**Results:**

We identified hospitalized and immunosuppressed patients with significantly higher levels of viral genetic diversity and variability. Further evaluation of serial samples revealed accumulated mutations associated with escape from neutralizing antibodies in a subset of the immunosuppressed patients treated with antiviral therapies. Interestingly, the accumulated viral mutations that arose in this early Omicron wave, which were not common in the patient viral lineages, represent convergent mutations that are prevalent in the later Omicron sublineages, including the XBB, BA.2.86.1 and its descendent JN sublineages.

**Conclusions:**

Our results illustrate the importance of identifying convergent mutations generated during antiviral therapy in immunosuppressed patients, as they may contribute to the future evolutionary landscape of SARS-CoV-2. Our study also provides evidence of a correlation between SARS-CoV-2 convergent mutations and specific antiviral treatments. Evaluating high-confidence genomes from distinct waves in the pandemic with detailed patient metadata allows for discerning of convergent mutations that contribute to the ongoing evolution of SARS-CoV-2.

**Supplementary Information:**

The online version contains supplementary material available at 10.1186/s12985-024-02378-y.

## Introduction

Since its initial emergence, severe respiratory syndrome coronavirus 2 (SARS-CoV-2), the virus responsible for the COVID-19 pandemic, has evolved into a series of variants that emerged sequentially and are characterized by distinct mutation profiles, from Alpha, Beta, and Delta variants to the later Omicron lineages [1]. The early stage of SARS-CoV-2 major variants evolution has been termed the first generation of variants of concern (VOCs) [2]. One main driver of this evolution has been hypothesized to be viral mutations generated during prolonged infections in immunosuppressed patients [3]. Indeed, multiple independent case studies of immunosuppressed patients revealed that viral mutations accumulated during persistent viral replication were consistent with those seen in the VOCs [4–8], highlighting the important role of viral replication in immunosuppressed patients in contributing to SARS-CoV-2 evolution. Starting from late 2022, the pandemic has been dominated by a series of Omicron sublineages, including descendants of BA.2 and recombinant viruses such as the XBB sublineage [2, 9]. These sublineages, representative of “second-generation variants,” are notable for a remarkably accelerated process of convergent evolution [2, 3]. However, the factors that are independently associated with this accelerated convergent evolution are not fully understood.

In this study, we deep sequenced viral RNA isolated from 192 patients and performed comparative bioinformatic analysis to: 1) identify patients with elevated levels of viral genetic diversity; 2) identify sites of convergent mutations in patients that arose in the first generation variants and became fixed in second generation variants of SARS-CoV-2; and 3) determine if specific therapies were associated with the selection of virus escape mutants and therefore likely contributed to the convergent evolution of SARS-CoV-2. We also compared the viral mutations identified in our patients to those reported either during in vitro drug selection studies or in case studies of patients with prolonged infections to identify sites of convergent mutations that contribute to virus evolution.

## Materials and methods

### Sample collection

All SARS-CoV-2 positive nasopharyngeal swab specimens were obtained at our medical center for clinical purposes, including those taken from patients who were hospitalized for COVID-19, immunosuppressed, and/or sampled multiple times, using a protocol approved by the Loyola University Health Sciences Campus Institutional Review Board (IRB #214,365). We sequenced 210 SARS-CoV-2 positive nasopharyngeal swab RNA samples from 192 patients. The majority of the samples were collected from November 2021 to November 2022, except for two samples (P1 and P2) that were collected in May and August 2021, respectively; P1 is a known Alpha sample that was used as a sequencing positive control and therefore was excluded in the analysis of viral mutations. Most patients were over 50 years old (median age male = 68; female = 67) and had received at least one dose of a COVID-19 vaccine (99%). Among these patients, 64% were hospitalized due to COVID-19, and 39% were immunosuppressed (Table 1). Information such as infected lineages, testing dates, patient age, gender, race, reason for immunosuppression, diabetes status, and SARS-CoV-2 vaccination were included in Supplemental Dataset 1.

**Table 1:** Summary of patient demographics (*n* = 192)

	N or Range	% or Median
Gender		
Male	102	53%
Female	90	47%
Age range (Median)		
Male	28–108	68
Female	28–95	67
Vaccination		
Vaccinated (≥ 1 dose)	190	99%
Unvaccinated or Unknown	2	1%
Immune status		
Immunosuppressed^a^	75	39%
Not Immunosuppressed	117	61%
COVID-related hospitalization		
Hospitalized	122	64%
Not hospitalized	70	36%
Diabetes mellitus		
Yes	105	55%
No	87	45%

^a^Immunosuppression due to active treatment for solid tumor and hematologic malignancies, recipients of solid organ transplant, patients with autoimmune diseases, or HIV infection as included in Supplemental Dataset 1

For patients who were sampled twice with near-full viral genome coverage, COVID-related medication history before and after the initial sampling event was also collected (Supplemental Table 1). We define immunosuppressed patients in our dataset as those with the following medical conditions: active treatment for solid tumor (*n =* 3) and hematologic malignancies (*n =* 3), receipts of solid-organ transplant (*n =* 63), patients with autoimmune diseases (*n =* 4), and advanced or untreated HIV-infected patients (*n =* 2). We performed binomial logistic regression analysis in R using the *glm()* function to evaluate the association between hospitalization status (i.e., hospitalized due to COVID-19 or not hospitalized) and factors including age, gender, diabetes status, immune status, and viral load (Ct values). Patient hospitalization status was associated with age (*p*-value = 0.003), male gender (*p*-value = 3.82 × 10^–4^), and immunosuppressed status (*p*-value = 0.03).

### Sample processing, extraction, and sequencing methods

Sample processing, RNA extraction, qPCR screening, and sequencing library prep methods were previously described [10]. Briefly, RNA was extracted from 200 μL of each sample using the MagMAX Pathogen RNA/DNA Kit (Applied Biosystems, Foster City, CA, USA) on a KingFisher Flex automated system (Thermo Fisher Scientific, Waltham, MA, USA). Viral concentrations were quantified by reverse transcription-quantitative PCR using the CDC N1 assay [11] (2019-nCoV RUO Kit, Integrated DNA Technologies, Coralville, IA, USA). A human RNase P assay (2019-nCoV RUO Kit) was performed with the same program as sample quality control. All samples with a cycle threshold (Ct) of < 33 were subjected to amplicon sequencing. A total of 210 RNA samples were sequenced using the NEBNext ARTIC SARS-CoV-2 FS Library Prep Kit for Illumina (E7658L, New England Biolabs, Ipswich, MA, USA) according to the manufacturer’s standard protocol with the ARTIC V4.1 primer panel (catalog #10,011,442, Integrated DNA Technologies, Coralville, IA, USA). Sequencing runs were performed at the Loyola Genomics Facility using an Illumina Miseq with 300-cycle V2 reagent kits.

### Sequencing data analysis

Raw reads were assessed for quality using FastQC [12], followed by adaptor trimming in cutadapt [13] and quality trimming in BBduk (http://jgi.doe.gov/data-and-tools/bb-tools/). Bwa-mem [14] was used for paired-end reads mapping to the Wuhan-Hu-1 reference genome (NCBI RefSeq accession NC_045512.2). Primer trimming was performed with iVar [15] using the bed file specific to ARTIC V4.1. Reads deduplication was then performed using Picard (http://broadinstitute.github.io/picard/) [16]. For all sequenced patient viral RNA samples (*n =* 210), an average genome breadth of coverage of 99.7% ± 0.4% (mean ± SD) was obtained with an average read depth of 1,132 ×  ± 337 × .

Viral mutations were called using iVar [10] with *p*-value < 0.05, a minimum read depth (*-m*) of 100, and a minimum frequency threshold (*-t*) of 0.02 and 0.5 for intra-host single nucleotide variant (iSNV) calling and major mutations calling, respectively. The results were converted to variant call format (VCF) files. Sites that may be artificially impacted by the ARTIC V4.1 primer panel were removed from the VCF file [17].

For iSNV identification, we set the threshold to be ≥ 2%, as this threshold has been proven to be confident excluding false positives such as those caused by sequencing errors [3, 16]. Samples were evaluated at 2%-95% frequencies (equal to or greater than 2% and less than 95% frequency) for the presence of true positive iSNVs but not lineage-defining (fixed) mutations [16, 18], and at 2%-50% frequencies (equal to or greater than 2% and less than 50% frequency) for low frequency iSNVs as an indication of viral genetic diversity in patient samples. The ratio of non-synonymous to synonymous substitutions (dN/dS) was identified using SnpEff [19].

Consensus genomes were generated using bcftools (v1.12) [20] based on the major mutation VCF files (≥ 50% frequency) with the exclusion of the low-coverage regions (< 10 reads), which were identified by bedtools [21]. A phylogeny tree using all 210 consensus genome sequences was generated using the Nextstrain [22] SARS-CoV-2-specific procedures and annotated using the interactive tree of life (iTOL) (https://itol.embl.de). Consensus genomes were analyzed through the Nextclade CLI [23] for PANGO lineage and private mutation information for each sample. Private mutations refer to viral mutations that are not commonly observed in the same SARS-CoV-2 lineage globally. These mutations, when their presence is confident (i.e., not caused by sequencing error, assembly error, etc.), can serve as indicators of viral genetic variability in comparison to the same lineages on the Nextclade reference tree. Here we term these private mutations as “non-lineage-defining mutations”, as they are distinct from the fixed mutations contributing to the lineage identification. A greater number of such non-lineage-defining mutations in a high-quality viral genome indicates a higher level of viral genetic variability on the consensus genome level compared to the same sublineage globally.

### Patient and community viral lineages comparison

To compare patient viral lineage trend with the local community, the “*getGenomicData*” function in the *outbreakinfo* R package [24] was used to access the SARS-CoV-2 lineage prevalence data in Illinois from November 1, 2021, to November 30, 2022. The community lineages across time were visualized using the R package *ggplot2* [25].

### Spike protein structure visualization

The structure of SARS-CoV-2 Omicron spike protein was accessed from the protein data bank (PDB, accession number 7T9J) and visualized with UCSF ChimeraX version 1.7 [26].

## Results

### Evaluating SARS-CoV-2 viral lineages in 192 patient samples

**Fig. 1:**
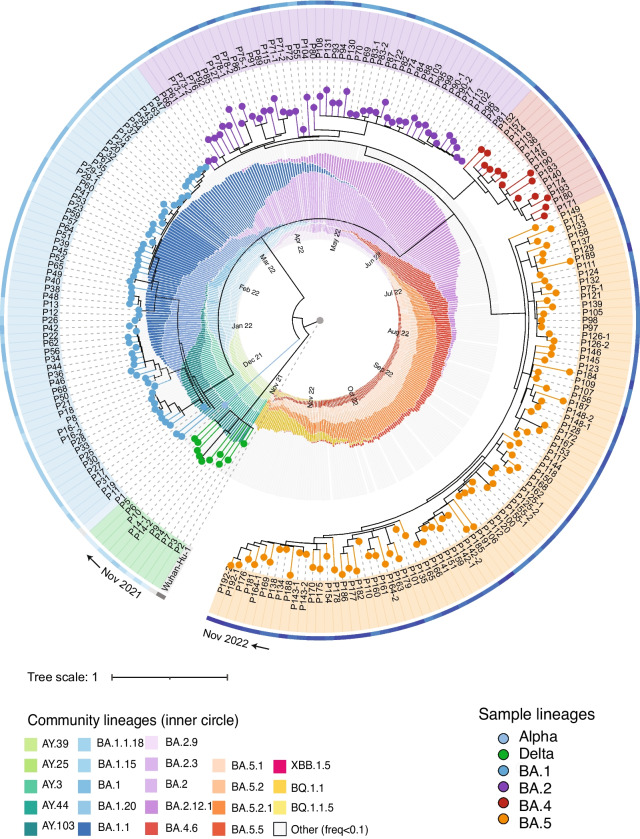
SARS-CoV-2 lineages identified in patient RNA samples (*n* = 210) and the comparison to lineages reported in the state of Illinois during the same period. Patient samples (phylogenetic tree) are annotated with different clade colors representing variants from Delta to Omicron BA.1 to BA.5 (except for one Alpha, P1, that was collected in May 2021 and serves as a positive control for sequencing). The outside color strip indicates the sample collection time from November 2021 (light blue) to November 2022 (dark blue). The inner ring shows lineages identified in Illinois with months annotated inside of the circle; sublineages > 10% frequency are shown in the representative color scheme, and lineages < 10% frequency are included in “other” in gray

We obtained 210 nasopharyngeal swab samples from 192 patients with the majority of the samples collected from November 2021 to November 2022. RNA was isolated and sequenced using the ARTIC V4.1 primer panel. The > 99% average genome breadth coverage and > 1,100 × average read depth allowed us to confidently identify the lineage of each patient sample. PANGO lineage analysis revealed a transition in variants from Delta (AY sublineages) to Omicron (BA.1, BA.2, BA.4, and BA.5 sublineages) from November 2021 to November 2022 (Fig. 1, Supplemental Dataset 1). This lineage transition is consistent with the community variants during the same period (Fig. 1). This broad representation of lineages allowed us to further investigate the level of intra-host single nucleotide variants (iSNVs) in these samples.

### Criteria for sample filtering for accurate iSNV identification

We use the level of iSNVs in each patient sample as an indication of the viral genetic diversity. To be confident in iSNV identification, we narrowed down patient samples to those of ≥ 99% genome breadth of coverage and ≥ 500 × average read depth (*n =* 195). To further assess possible impacts from sample quality on iSNV identification, especially on low frequency iSNV, we examined the correlation between viral loads (here Ct values) and iSNV numbers in patient samples (Figure S1). We found that the numbers of all frequency (2–95%) and low frequency (2–50%) iSNVs were positively correlated with Ct values (Figure S1AB; Pearson’s *r* = 0.421, *p*-value = 9.2 × 10^–10^ for 2–95% iSNVs, Pearson’s *r* = 0.441, *p*-value = 1.1 × 10^–11^ for 2–50% iSNVs). This significant positive correlation was also observed in 2–5% frequency iSNVs in our dataset (Figure S1C). However, for higher frequency (50–95%) iSNVs, the correlation between mutation numbers and Ct was not observed (Figure S1D, Pearson’s *r* = -0.057, *p*-value = 0.427). Particularly, samples with Ct > 30 showed significantly higher numbers of iSNVs than those with Ct ≤ 30 in all frequency and low frequency iSNVs (Wilcoxon test, *p*-value = 2.4 × 10^–5^ for 2–95% iSNVs, *p*-value = 1.6 × 10^–6^ for 2–50% iSNVs). This suggests a higher possibility of false positive mutations associated with low viral RNA input, especially in low frequency mutations that are strong indicators of within-host viral genetic diversity. To maximize the accuracy in the downstream analysis of iSNVs in patient groups, we analyzed samples with ≥ 99% genome breadth of coverage, ≥ 500 × average read depth, and with a Ct value ≤ 30 (*n =* 176).

### Levels of SARS-CoV-2 genetic diversity and variability are higher in hospitalized and immunosuppressed patients

**Fig. 2:**
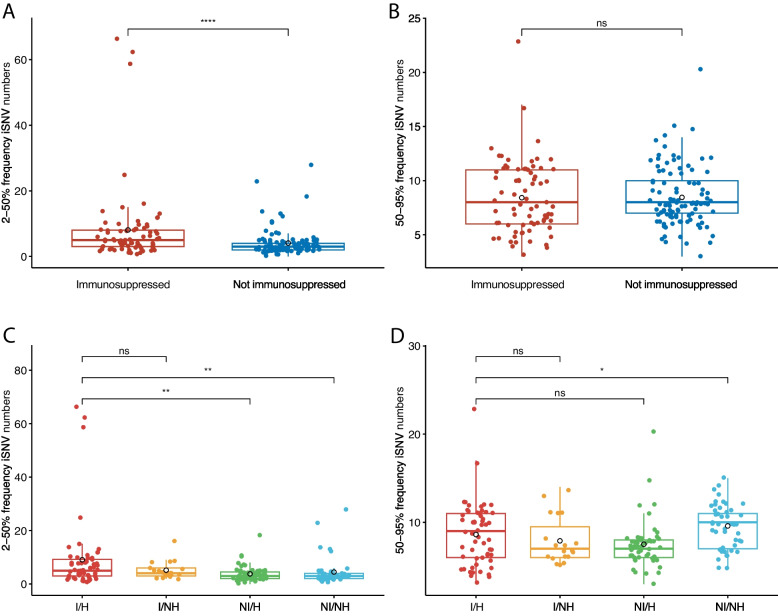
Levels of iSNVs are higher in immunosuppressed and hospitalized patients. **A** 2–50% frequency iSNVs in patients of different immunity status. **B** 50–95% frequency iSNVs in patients of different immunity status. **C** 2–50% frequency iSNVs in patients of different status of immunity and hospitalization. **D** 50–95% frequency iSNVs in patients of different status of immunity and hospitalization. The horizontal lines in the box plot represent for 25%, 50% and 75% data percentile. The mean value of each group is shown in black hollow circle. Statistics are performed using the Wilcoxon test, with the *p*-value significance shown in each comparison. I/H, immunosuppressed and hospitalized; I/NH, immunosuppressed and not hospitalized; NI/H, not immunosuppressed and hospitalized; NI/NH, not immunosuppressed and not hospitalized

To evaluate the levels of genetic diversity in the samples, we assessed levels of iSNVs based on read frequencies in the chosen patient samples. We analyzed the iSNVs at different frequencies: 2–95% frequency to capture all iSNVs; 50–95% frequency to assess high frequency iSNVs; and 2–50% frequency to assess low frequency iSNVs. We found a significantly higher average number of iSNVs in immunosuppressed patients (Figure S2A; Wilcoxon test, *p*-value = 0.008). When we grouped patients into different immunity and hospitalization statuses, we found that the immunosuppressed and hospitalized patients had the highest average number of all iSNVs (Figure S2B). We then investigated the levels of low frequency (2–50%) iSNVs, because such iSNVs are a strong indication of viral genetic diversity in the viral replication process [3, 16]. We found that immunosuppressed patients had significantly higher numbers of low frequency iSNVs compared to the non-immunosuppressed group (Fig. 2A; *p*-value = 4.9 × 10^–5^). Furthermore, the immunosuppressed and hospitalized patients had the highest average number of low-frequency iSNVs in all subgroups (Fig. 2C), and this is statistically significant (*p*-value = 0.001 and 0.002, respectively). The low frequency iSNV level differences are significant even when the three potential outliers are removed (Figure S3). Evaluating the ratio of non-synonymous to synonymous substitutions (dN/dS) in these low frequency mutations revealed a higher ratio in immunosuppressed patients (*p*-value = 0.005; Figure S4). In contrast, when comparing levels of high frequency (50–95%) iSNVs in the same groups, we did not detect a difference between immunosuppressed and non-immunosuppressed patients (Fig. 2B). Further examination of these iSNVs in the four subgroups of immunity and hospitalization did not show a higher level of high frequency iSNVs in the immunosuppressed and hospitalized patient group (Fig. 2D). Overall, these results indicate that the elevated viral genetic diversity in our immunosuppressed and hospitalized patient samples is likely driven by replication that generates low frequency mutations (iSNVs).

In addition to the iSNVs analysis described above, we investigated the non-lineage defining mutations to further understand the viral genetic variability between the patient’s viral genomes and the nearest neighbor PANGO lineage. This analysis compares the patient genome sequence to the nearest neighbor on the Nextclade reference tree (see Materials and methods). The analysis reveals the genetic variability of these viral genomes, while taking into consideration viral mutations that are fixed in the lineage specific to each sample. We found a slightly higher level of non-lineage-defining mutations in the immunosuppressed patients (mean = 4.2 mutations/genome) than in the non-immunosuppressed patients (mean = 3.6/genome), although this was not statistically significant (Figure S5A). Further analysis of the data revealed the highest average number of non-lineage-defining mutations in immunosuppressed and hospitalized patients and the lowest in the non-immunosuppressed outpatients (Figure S5B). Particularly, the patients that had the highest level of non-lineage defining mutations (> 10/genome, *n* = 8), including three samples with high iSNV levels (Fig. 2A; P14, *n* = 66; P29, *n* = 59; P190, *n* = 25), were all immunosuppressed and hospitalized (Figure S5A, black dashed box). Taken together, these results indicate a greater genetic diversity in the immunosuppressed and hospitalized patients, and that a subset of these patients had higher levels of genetic variability on the consensus genome level compared to the nearest neighbor on the Nextclade reference tree.

### A subset of immunosuppressed patients accumulated multiple major mutations over time with a focus on ORF1a and spike convergent mutation sites

**Fig. 3:**
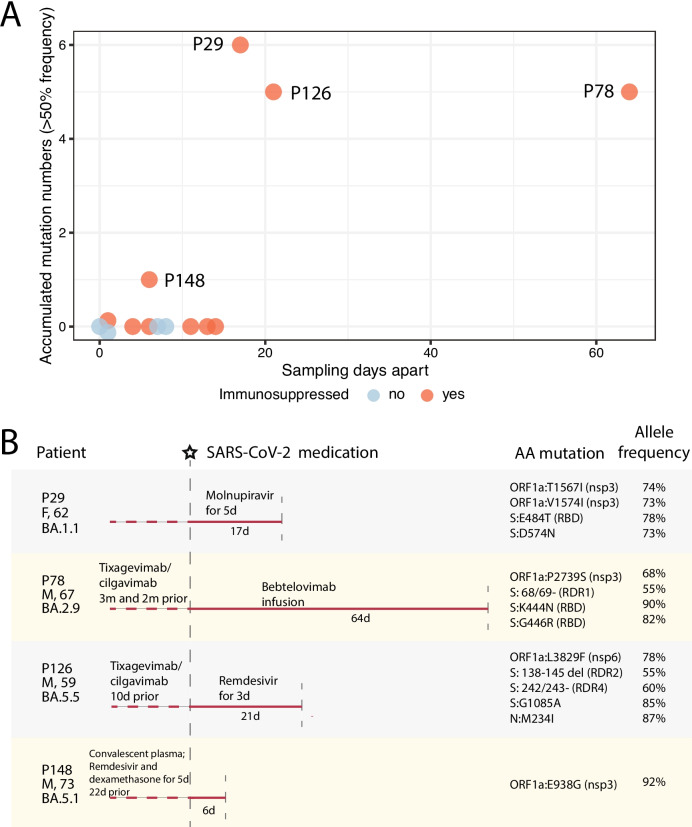
Accumulation of mutations in patients over time, as revealed by serial sampling and sequencing. **A** The number of accumulated nucleotide mutations detected in 14 repeatedly sampled patients. Orange dots represent immunosuppressed patients, and blue dots represent non-immunosuppressed patients. The y-axis shows the number of patients’ mutations changed between two samplings, and the x-axis shows sampling intervals (days). **B** Schematic diagram of SARS-CoV-2 treatment history and viral mutation accumulation in five immunosuppressed patients. Patient ID, gender, age, and infected viral sublineages are shown on the left side. The vertical dashed gray lines represent the 1st (starred) and 2nd sampling events, with days apart annotated under the line. Medication before and after the 1st sampling event is shown above the line. Amino acid mutations accumulated between two sampling events and their allele frequencies are shown on the right

With the observation of the higher level of genetic diversity and variability in immunosuppressed and hospitalized patients, we hypothesized that the viral evolution in those patients may be associated with viral replication in the absence of a strong adaptive immune response and selective pressure from COVID-related antiviral treatments. We therefore examined the consensus genome sequences and read alignment of a subset of patients who were sampled twice (*n* = 15) for their mutation changes between the sampling events. Among these, one patient (P75) was sampled more than three months apart and was found infected with two different sublineages (BA.2.3 in April 2022 and BA.5.5 in August 2022) and was therefore excluded in the subsequent analysis. The other 14 patients were all identified as having the same sublineage in two samplings (average of 12 days apart), allowing for analysis of viral evolution in these patients. We identified three distinct Omicron sublineages in these patients (BA.1, BA.2, and BA.5, Supplemental Table 1). Among the 14 patients, 10 were immunosuppressed (Fig. 3A), including P14 and P29 which had high levels of genetic variability. Four out of the ten had accumulated major mutations between the two samplings (nucleotide mutation number 4.3 ± 2.2, mean ± SD; sampling intervals 27.0 ± 25.5 days, mean ± SD). We identify nonsynonymous mutations that progressed from < 50% read frequency in the 1st sampling to ≥ 50% read frequency in the 2nd sampling as accumulated mutations. Three patients showed evidence of multiple accumulated mutations (P29, P78, and P126) and one had evidence of a single mutation (P148) (Fig. 3A, Supplemental Table 1). Non-immunosuppressed patients (*n* = 4) had no detectable consensus genome changes (sampling intervals 4 ± 4.1 days) (Fig. 3A). The three immunosuppressed patients with multiple mutations all had at least two weeks apart between the sampling events. These results document that immunosuppressed patients accumulate viral mutations during ongoing virus replication.

**Fig. 4:**
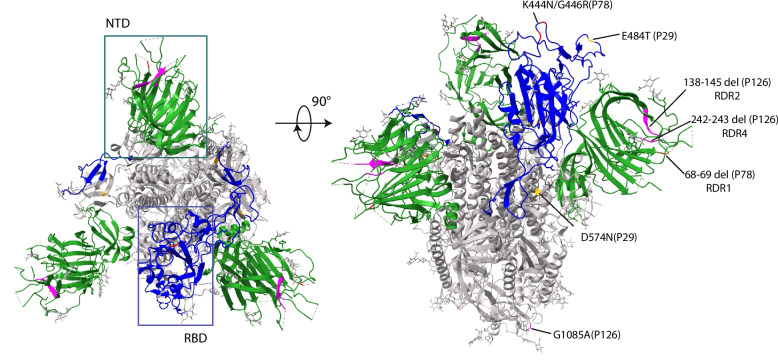
The SARS-CoV-2 Omicron spike structure (PDB 7T9J) top (left) and side (right) view with the accumulated mutations in three immunosuppressed patients. The NTDs in the trimer are shown in green and the RBD is shown in blue as indicated in the boxes on the left panel. The spike deletions and substitutions in P29, P78, and P126 are labeled and marked on the structure view in yellow, red, and pink colors, respectively

The accumulated amino acid mutations (*n* = 14) in the four immunosuppressed patients were mostly in the ORF1a (nsp3 and nsp6) and the spike regions (Table 2) and were all non-lineage-defining mutations as identified by Nextclade (i.e., mutations not commonly found in the sample’s lineages globally). We asked if the mutations detected in these patients were random or were examples of sites of convergent evolution that were also detected in later variants. Table 2 shows the information on the corresponding amino acid mutations and their convergent mutations in the literature, and in variants and lineages up to date (accessed from the GISAID platform using the *outbreakinfo* R package). Most of the accumulated amino acid mutations identified in our study, particularly those in the spike gene, are consistent with those described in case studies of prolonged infection in immunosuppressed patients, and in vitro studies of antibody neutralization. Interestingly, these early-wave Omicron-infected immunosuppressed patients share the same sites of mutation as those reported in many later variants, including the S: K444 and ORF1a:L3829F sites that are fixed in multiple later Omicron sublineages and S:G446 in the recent BA.2.86.1 and its descendent JN sublineages (see Table 2 for the presence of all patient convergent mutations fixed in early or late SARS-CoV-2 lineages). Similarly, deletions in the spike NTD recurrent deletion regions (RDRs) [27] identified in two patients (P78 and P126; RDR1, RDR2, and RDR4) are also found in patient case studies and early or late sublineages (Table 2). Interestingly, these RDR deletions in our patients were present in lineages distinct from previous reports, suggesting adaptative convergent evolution of SARS-CoV-2 in these patients. Furthermore, when mapping the spike mutations in our patients onto the SARS-CoV-2 Omicron spike structure (Fig. 4), these mutations are all located on the surface of the NTD and RBD domains, as well as in the S2 connector domain (CD), indicating their potential structural and functional impacts, such as escaping from neutralizing antibodies. Taken together, our observations indicated that the accumulated mutations (deletions and substitutions) are independent adaptations to the viral replication process in these early Omicron-infected immunosuppressed individuals and correspond to the later convergent evolution genetic landscape of SARS-CoV-2.

**Table 2:** Information on mutations identified in immunosuppressed patients in this study and in other studies and SARS-CoV-2 lineages, consistent with sites of convergent evolution

Patient ID	Accumulated mutations	Relevant immunosuppressed patient case studies	Relevant mAb and/or antiviral studies^a^	Convergent mutations fixed in variants^b^
P29 BA.1.1	ORF1a:T1567I (nsp3)	This study	N/R	Kappa
	ORF1a:V1574I (nsp3)	This study	N/R	/
	S:E484T (RBD)	Halfmann et al. 2023 [28] reported in a patient treated with bamlanivimab	Resistance to bamlanivimab [28]	Beta, Gamma, and all Omicron sublineages (convergent mutations: S: E484K/A/R)
	S:D574N	This study	Among the most recurrent G-A mutations related to molnupiravir [29]	/
P78 BA.2.9	ORF1a:P2739S (nsp3)	This study	N/R	/
	S: 68/69-(NTD; RDR1)	S:69/70- convergent deletion mutation by Kemp et al. 2021 [7] and Sonnleitner et al. 2022 [30], etc.	S:69/70- increases viral entry efficiency and infectivity [31]	Alpha, BA.1, BA.3, BA.4, BA.5, and CK, BE, BF, BQ.1 sublineages (convergent mutation: S:69/70-)
	S:K444N (RBD)	Ordaya et al. 2023 [32] on reduced susceptibility to bebtelovimab	In vitro confirmed sites confer a reduction in susceptibility to bebtelovimab [33, 34]	Later Omicron BQ.1, CH.1, BE.9, BR.1, CL.1, CK, CM, DL.1 sublineages (convergent mutations: S: K444T/M/N/R)
	S:G446R (RBD)	Convergent mutation reported by Ordaya et al. 2023 [32] on reduced susceptibility to bebtelovimab	In vitro confirmed sites confer a reduction in susceptibility to bebtelovimab [33]	Omicron BA.1.1, BA.1.12.1, BA.2.75, BA.5.2.43, and later BN, BM, CH.1, XBB (e.g., XBB.1.^★^, EG, FL, FK, FU, GK, HK, HV), XBC.1.^★^, XBF, BA.2.86.1 and its descendent JN.^★^ sublineages (convergent mutations: S: G446S/D)
P126 BA.5.5	ORF1a:L3829F (nsp6)	Morita et al. 2023 [35]	N/R	Later Omicron BQ.1, XBB.2.3.8, and XBB.1.16 sublineages
	S: 138/145- (NTD, RDR2)	Convergent deletion mutations by Hensley et al. [36] and Sonnleitner et al. 2022 [30]	Y144- and similar deletions confer to resistance to NTD-directed mAbs [27, 37]	Alpha, Omicron BA.1, and later BQ.1, XBB (e.g., XBB.1.^★^, EG, FL, FU, GK, HK, HV), XBC.1.^★^, BA.2.86.1 and its descendent JN.^★^ sublineages (convergent mutations: S:Y144- / 144/145- / 143/145-)
	S: 242/243- (NTD, RDR4)	Convergent deletion mutation by Nussenblatt et al. 2022 [38]	243/244- and similar deletions confer to resistance to NTD-directed mAbs [27]	Beta and later Omicron XBC.1.3 lineages (convergent mutations: S:243/244- / 241/243-)
	S:G1085A (S2 CD)	This study	N/R	/
	N:M234I	Díaz et al. 2021[39], Shoji et al. 2022 [40]	N/R	B.1 lineage
P148 BA.5.1	ORF1a:E938G (nsp3)	This study	N/R	/

^a^*N/R* not previously reported

^b^Variants and sublineages reported here are of > 90% frequency of the convergent mutations and > 1,000 counts in the GISAID database as of January 26, 2024; data was queried using the *outbreakinfo* R package

### Specific therapies were associated with the accumulation of convergent spike mutations in immunosuppressed patients

To understand possible factors associated with the occurrence of these convergent mutations in the repeatedly sampled immunosuppressed patients, particularly those who accumulated multiple mutations, we examined their related COVID-19 treatment history before and after the initial sampling events (Fig. 3B, Supplemental Table 1). We note that all immunosuppressed patients (*n* = 10) were treated with immunosuppressants, such as mycophenolate mofetil, prednisolone, and tacrolimus.

For non-immunosuppressed patients (*n* = 4), two had no reported treatments, and the other two (P90 and P125) had remdesivir and/or nirmatrelvir/ritonavir treatment but with short sampling intervals (≤ 1 day). For immunosuppressed patients without major mutation accumulation (*n* = 6), two had antiviral treatment history (P14, casirivimab/imdevimab and remdesivir 22 days before the 1st sampling and no treatment between a 11-day sampling interval; P143, nirmatrelvir/ritonavir and remdesivir between a 4-day sampling interval). For immunosuppressed patients who had mutations (*n* = 4), multiple antiviral treatment histories were reported. P78 and P126, who were treated with both small molecule inhibitor antivirals (e.g., remdesivir) and mAbs (e.g., tixagevimab/cilgavimab and bebtelovimab), showed mutations in ORF1a (nsp3 and nap6, respectively) and spike genes, including the spike RDR deletions (Fig. 3B). The RDR2 and RDR4 deletions in P126 are in two adjacent flexible loops in spike NTD (Fig. 4), suggesting that they may confer conformational changes in this domain to facilitate escape from neutralizing mAbs. The two spike RBD antigenic substitutions (K444N and G446R) in P78 are located in corresponding spike epitopes binding to tixagevimab/cilgavimab and bebtelovimab [41], the treatments of this patient, suggesting a selective pressure from the mAb treatments on the intra-host viral evolution. Interestingly, P29 had a treatment history of molnupiravir and showed multiple substitutions (*n =* 6) on ORF1a and spike, including the S:E484T and S:D574N mutations. Molnupiravir mainly disrupts SARS-CoV-2’s nascent RNA strand synthesis or positive-sense genome synthesis by incorporating itself as an analogue of cytosine (C) and therefore leads to guanine (G) to adenine (A) or cytosine (C) to uracil (U) mutation [29]. Both spike amino acid mutations in P29 were derived from the G to A nucleotide mutation, strongly supporting the correlation between viral mutation accumulation and the molnupiravir treatment. P148 only had convalescent plasma and remdesivir treatments 22 days prior to the 1st sampling and accumulated an nsp3 mutation (ORF1a:E938G) during the samplings. These developed substitutions in the four patients all had allele frequencies between 73 to 92% (Fig. 3B), and were all derived from < 2% frequency in the 1st sampling (i.e., mutations were not called at 2% cutoff), except for the single mutation in P148 that was progressed from 25% frequency in the 1st sampling. Taken together, these results strongly support the selection pressure from antiviral treatments in immunosuppressed patients during ongoing viral replication and the role of antiviral therapies in contributing to the selection of SARS-CoV-2 convergent evolutionary sites in this population.

## Discussion

Our study evaluating SARS-CoV-2 evolution in 192 patients throughout a year with comparison to the community variants strongly supports the central role of persistent infection in the generation of intra-host SARS-CoV-2 mutations in immunosuppressed patients, particularly those undergoing antiviral treatments. We found that the viral lineages in our main patient cohort are consistent with the community variants (Fig. 1). However, the notably higher level of viral genetic diversity and variability in some hospitalized and immunosuppressed patients (Fig. 2) indicates their potential for acting as independent “reservoirs” for convergent mutations. Importantly, we show that during prolonged virus replication, immunosuppressed patients tend to accumulate host-adaptive mutations (Fig. 3), and that such viral mutation accumulation is not rare (in 4/10 repeatedly sampled immunosuppressed patients). The mutations are likely associated with resistance to mAbs and other antiviral treatments (Fig. 3B). These results strongly support the idea that persistent infection in immunosuppressed individuals may serve as the reservoir for SARS-CoV-2 immune-escaping mutations and contribute to the ongoing evolutionary landscape of SARS-CoV-2 [3–8, 30, 38].

Our study aligns with prior findings highlighting the potential functional impacts of convergent evolution in the SARS-CoV-2 spike protein. The spike NTD region is highly plastic and very likely to have recurrent deletions [27, 42]. The RBD substitutions at positions R346, K444, and L452 are associated with escape from neutralizing antibodies [43]. Antigenic substitutions and RDR deletions in spike NTD and RBD in our study, which are mostly located on the surface of the spike protein (Fig. 4), are associated with antibody escape in other patient studies and/or in vitro neutralizing antibody studies (Table 2), suggesting functional roles of these mutations in response to factors like host immunity and mAb-like treatments. Further, the substitutions in the immunosuppressed patients in our study are within the “evolutionary hotspots” of the SARS-CoV-2 RBD region; mutations such as G446X are ubiquitous in multiple later Omicron sublineages, including the XBB recombinants [44], BA.2.86.1 and its descendent JN lineages (Table 2), supporting the importance of these convergent mutations in viral evolution. Our study reports independent observations of convergent mutations in the spike protein in immunosuppressed patients undergoing antiviral therapies, illustrating the potential role of antiviral therapy in this vulnerable population in driving SARS-CoV-2 convergent evolution.

Our study also contributes to the knowledge of SARS-CoV-2 convergent mutations correlating with specific antiviral treatment regimens in immunosuppressed patients. The mutations accumulated in our immunosuppressed patients occurred predominantly in ORFla (nsp3 and nsp6, proteins that contribute to the formation and organization of the double membrane vesicles for viral replication) and spike genes, indicating the mutations correlated with the viral replication process under antiviral treatments. Our results from patients in the early Omicron wave are consistent with the findings from other individual case studies. For example, a case report of an immunosuppressed patient treated with remdesivir, dexamethasone, and convalescent serum also confirmed RDR2 deletions [27, 36], similar to P126 in our study who also had remdesivir and mAb treatments; Ordaya et al. reported mutations in S:K444 and S:G446 sites in response to bebtelovimab treatment in immunosuppressed patients, similar to P78. Specifically, P29 who had only molnupiravir treatment showed multiple nsp3 and spike substitutions that were caused by G to A or C to T nucleotide changes, which corresponds to the antiviral mechanisms of molnupiravir [29, 45]; this includes G23012A that led to the double codon mutation S:E484T, a derivative of E484A in the 1st sampling. Recently, E484T has been reported by Halfmann et al. [28] in a persistently infected immunosuppressed individual in response to mAb treatment (bamlanivimab), where the mutation also progressed from E484A. Together with our study, these findings support the concept that the convergent immune-escaping mutations could be derived from different types of antiviral therapies, and highlight the importance of delineating the correlation between antiviral treatment and immune-escape viral mutations. More reports of SARS-CoV-2 surveillance in immunosuppressed patients and their detailed treatment schemes are needed.

Our study has limitations. This study analyzed a relatively small number of samples obtained from one clinical center during a period of rapid change during the pandemic. The patient samples were obtained from individuals presenting with a variety of pre-existing conditions which may impact on disease progression.

## Conclusions

This study provides evidence of SARS-CoV-2 convergent evolution in immunosuppressed patients undergoing antiviral therapies. Our findings highlight the need for a better understanding of associations between viral mutations and specific antiviral therapies, which will ultimately lead to better strategies to limit virus replication and reduce the accumulation of novel mutations.

### Supplementary Information

**Supplementary Material 1.**

**Supplementary Material 2.**

## Data Availability

All sequencing data were uploaded to the National Center for Biotechnology Information (NCBI) Sequence Read Archive (SRA) under the Bioproject PRJNA1038785.

## Abbreviations

iSNV: Intrahost single nucleotide variant
mAb: Monoclonal antibody
NTD: N-terminal domain
RBD: Receptor-binding domain
SARS-CoV-2: Severe acute respiratory syndrome coronavirus 2
VCF: Variant call format
VOC: Variant of concern
