# Supplementary material for: Evidence of SARS-CoV-2 convergent evolution in immunosuppressed patients treated with antiviral therapies
Source: Virol J. 2024 May 7;21:105. doi: 10.1186/s12985-024-02378-y (PMC11075269; doi:10.1186/s12985-024-02378-y)
Supplement: Supplementary file 2 — Supplementary Material 2. [file 12985_2024_2378_MOESM2_ESM.pdf]

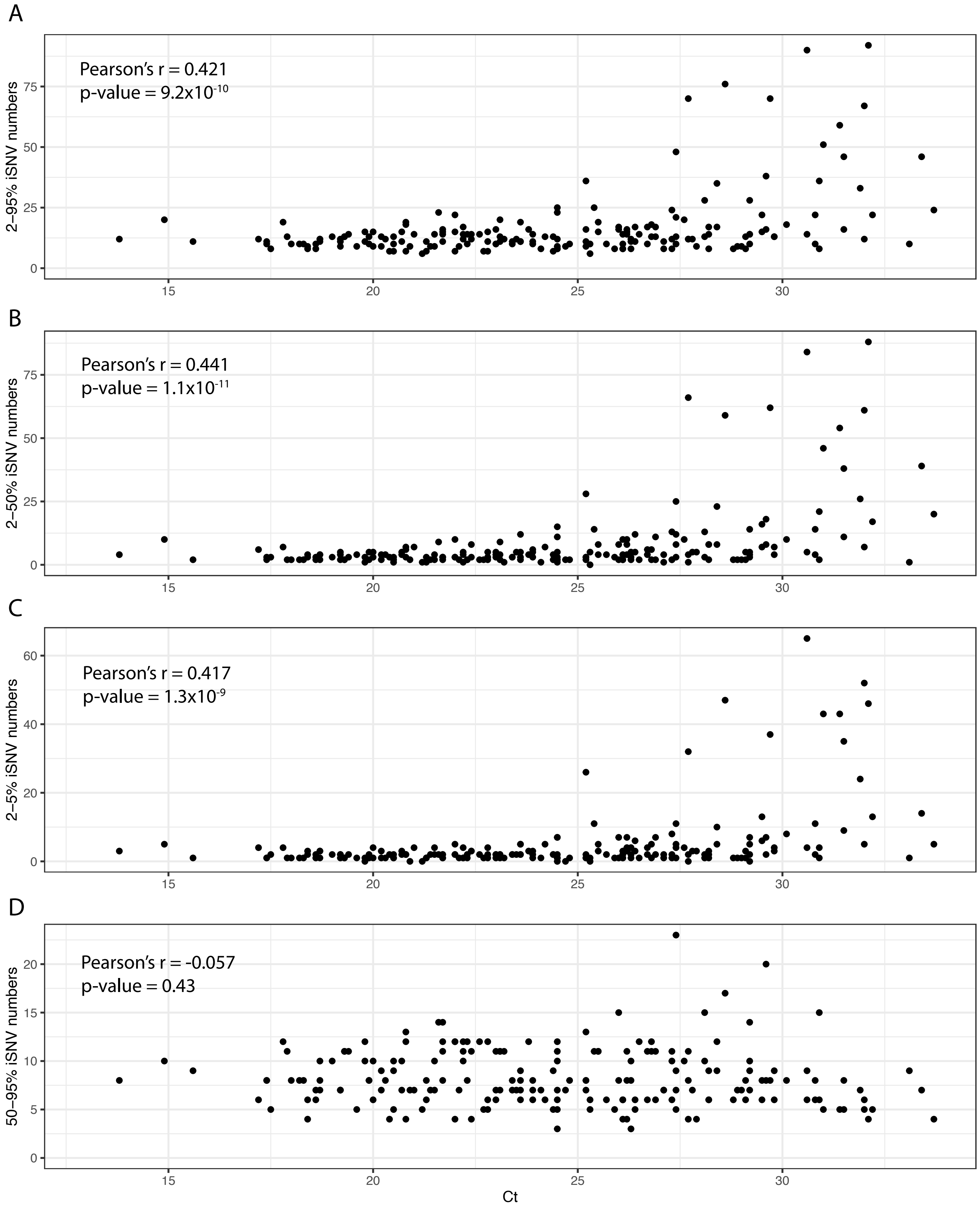

Figure S1. Correlation between iSNV numbers and Ct values of patient samples. The numbers of iSNVs are assessed based on A). 2-95% frequency, B). 2-50% frequency, C). 2-5% frequency, and D). 50-95% frequency. Pearson's coefficient and the corresponding p-values are shown in the upper left corner of each plot.

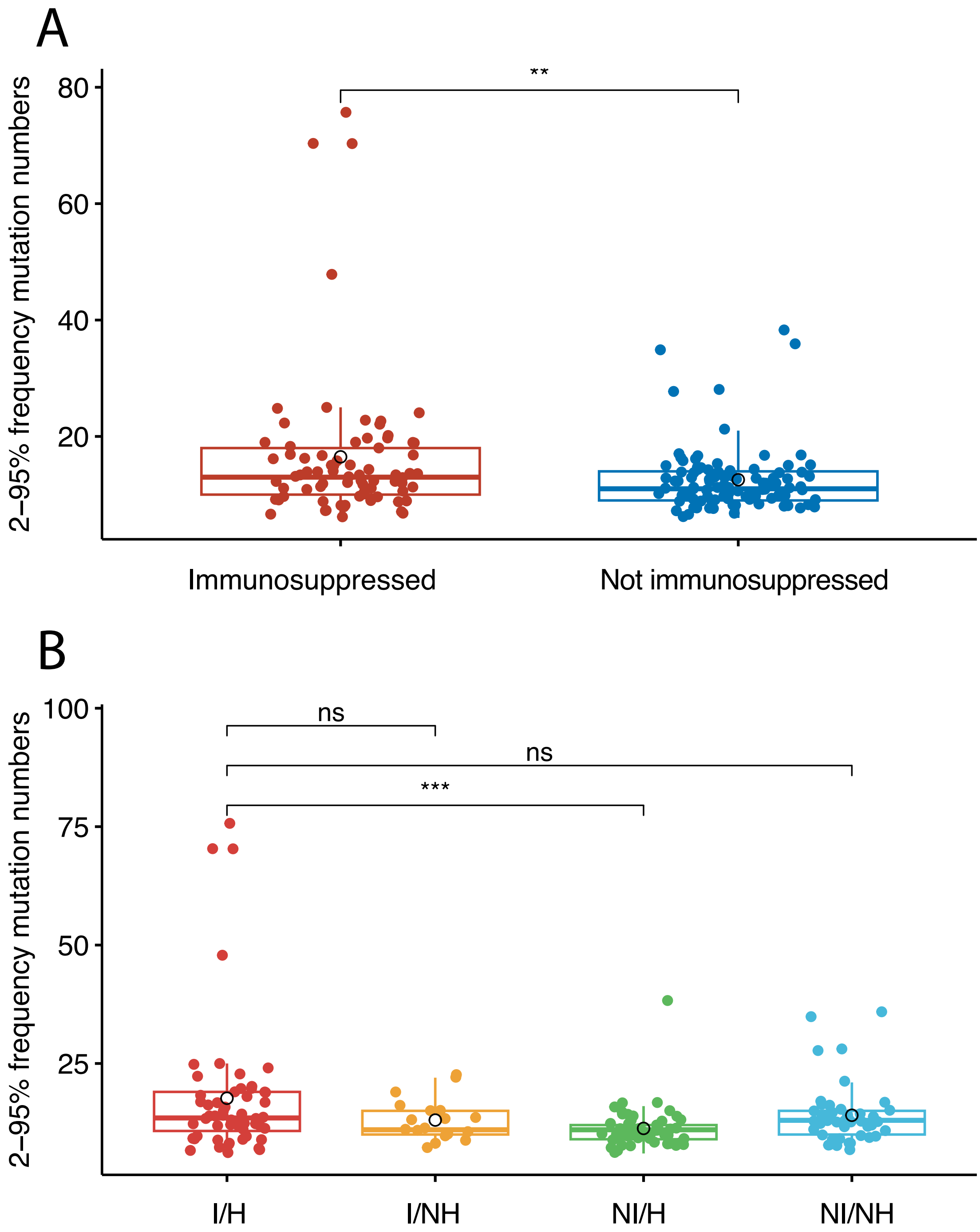

Figure S2. Levels of all iSNVs in patient groups. A). 2-95% frequency iSNVs in patients of different immunity status. B). 2-95% frequency iSNVs in patients of different status of immunity and hospitalization. The horizontal lines in the box plot represent for 25%, 50%, and 75% data percentile. The mean value of each group is shown in black hollow circle. Statistics are performed using the Wilcoxon test, with the p-value significance shown in each comparison. I/H, immunosuppressed and hospitalized; I/NH, immunosuppressed and not hospitalized; NI/H, not immunosuppressed and hospitalized; NI/NH, not immunosuppressed and not hospitalized.

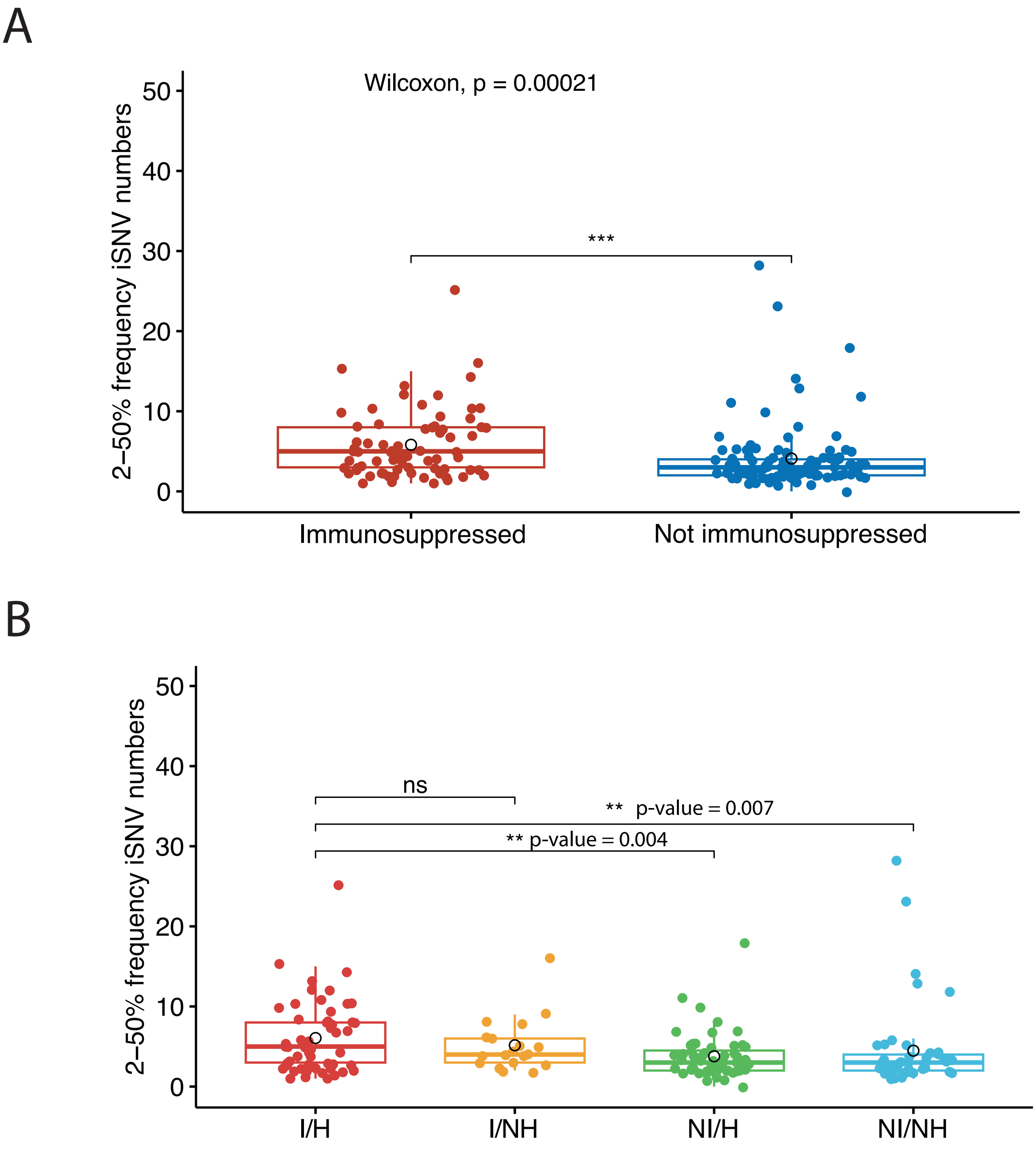

Figure S3. Levels of low frequency iSNVs in patients without the three patients who had the highest numbers (potential outliers). A). 2-50% frequency iSNVs in patients of different immunity status without the three outliers. B). 2-50% frequency iSNVs in patients of different status of immunity and hospitalization without the three outliers. The horizontal lines in the box plot represent for 25%, 50%, and 75% data percentile. The mean value of each group is shown in black hollow circle. Statistics are performed using the Wilcoxon test, with the p-value significance shown in each comparison.

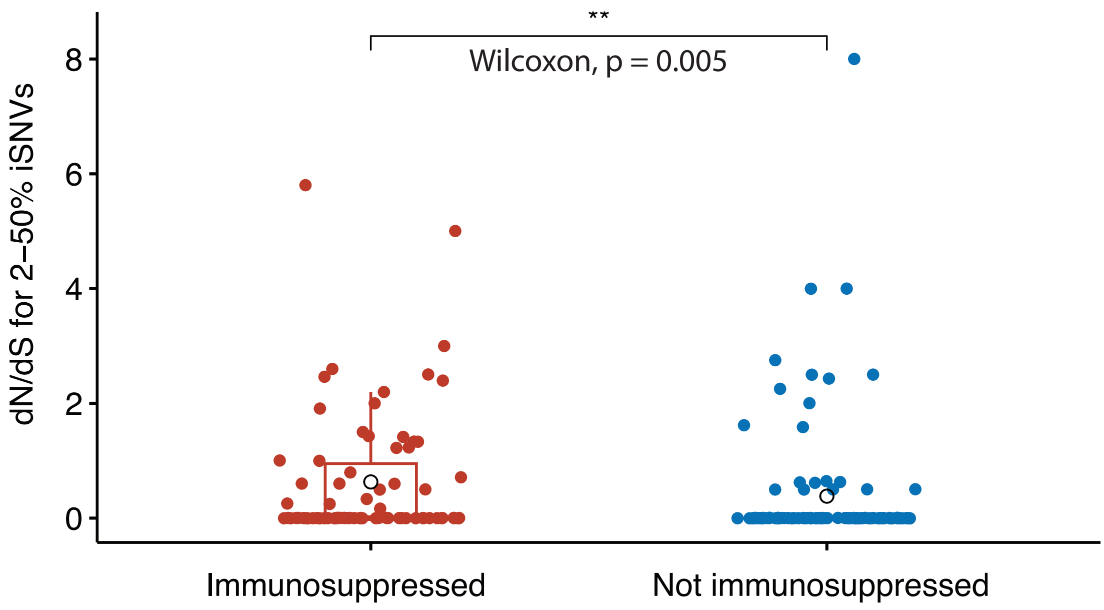

Figure S4. The ratio of non-synonymous to synonymous substitutions (dN/dS) in the low frequency mutations in immunosuppressed and not immunosuppressed patients (n=176). The mean value of each group is shown in black hollow circle. Statistic analysis is performed using the Wilcoxon test with the p-value significance shown. Samples with the ratio = 0 are those that have no identified non-synonymous and/or synonymous low frequency mutations.

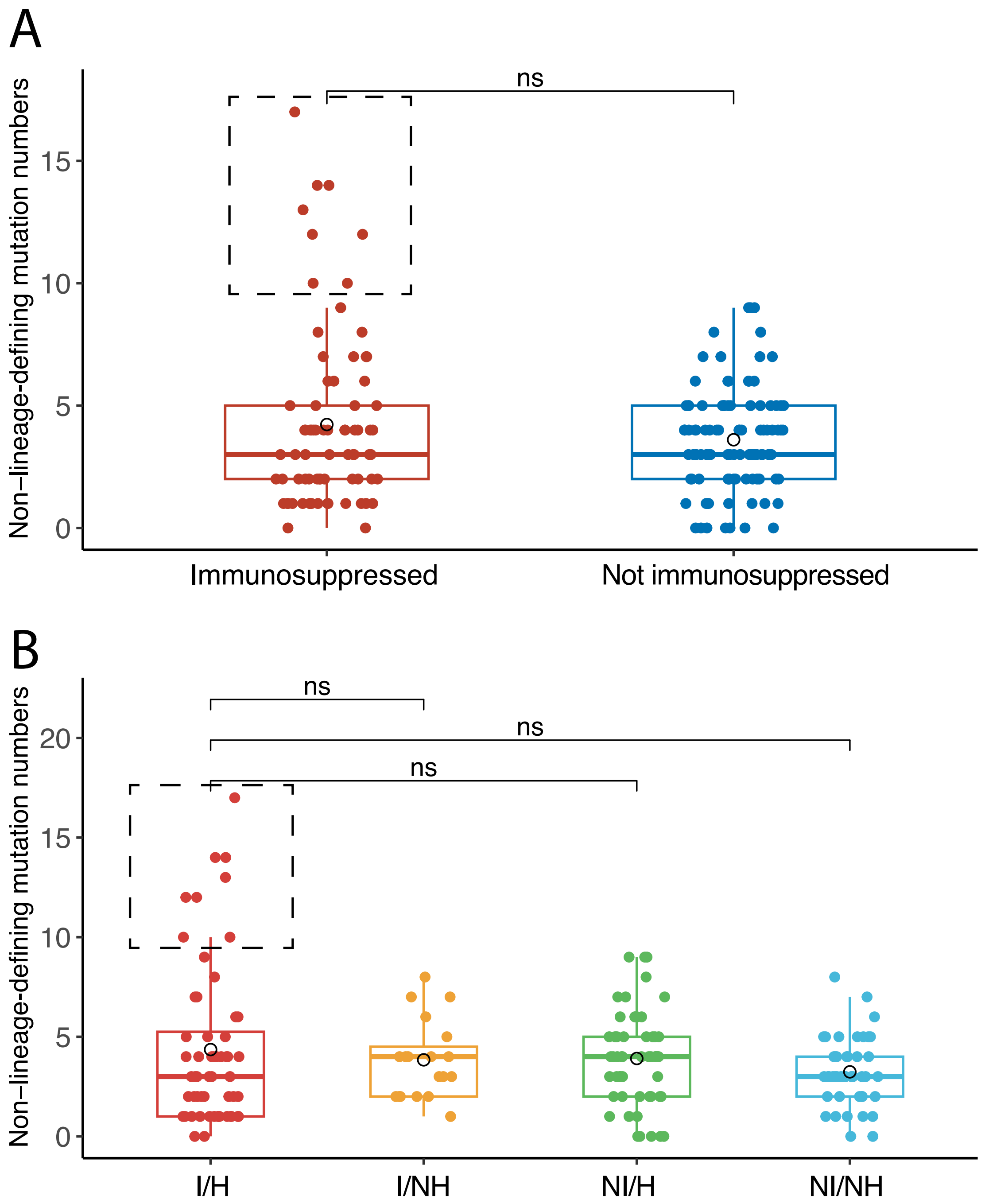

Figure S5. Levels of non-lineage-defining mutations in patient groups. A). Levels of non-lineage -defining mutations in patients of different immunity status. B). Levels of non-lineage-defining mutations in patients of different status of immunity and hospitalization. Samples in the black dashed box have more 10 or more lineage-defining mutations. The horizontal lines in the box plot represent for 25%, 50% and 75% data percentile. The mean value of each group is shown in black hollow circle. Statistics are performed using the Wilcoxon test, with the p-value significance shown in each comparison. I/H, immunosuppressed and hospitalized; I/NH, immunosuppressed and not hospitalized; NI/H, not immunosuppressed and hospitalized; NI/NH, not immunosuppressed and not hospitalized.

Supplemental Table 1. Information on the 14 repeatedly sampled patients in this study and their accumulated viral mutations.

Patient ID	Age/ Sex	COVID-related Hospitali-zation	Immuno-suppressed	Reason for immuno-suppression*	SARS-CoV-2 Lineage	1st sampling date	Sampling interval (days)	SARS-CoV-2 treatment before 1st sampling	SARS-CoV-2 treatments between samplings	Developed mutations >50% frequency (NT)	Developed mutations >50% frequency (AA)	Immunosuppressive medication	Other comorbidities**
P29	62/F	yes	yes	AML	BA.1.1	01/15/22	17	/	Molnupiravir for 5 days	T168C, C4965T, G4985A, C12784T, G23012A , G23282A	ORF1a:T1567I (nsp3), ORF1a:V1574I (nsp3), S:E484T (RBD), S:D574N	Decitabine, venetoclax	/
P78	67/M	hospitalized for non-COVID reason	yes	SOT-lung	BA.2.9	05/04/22	64	Tixagevimab/cilgavimab 2 months and 3 months prior, respectively	Bebtelovimab infusion	C186T, C8480T, 21763-21768 del, G22894T, G22898C	ORF1a:P2739S (nsp3), S: 68/69- (NTD; RDR1), S:K444N (RBD), S:G446R (RBD)	Mycophenolate mofetil, prednisolone, tacrolimus	DM, CKD
P126	59/M	hospitalized for non-COVID reason	yes	SOT-kidney	BA.5.5	07/14/22	21	Tixagevimab/cilgavimab; 10 days prior	Remdesivir for 3 days	C11750T, 21972-21995 del, 22289-22294 del, G24816C, G28975T	ORF1a:L3829F (nsp6), S: 138-145del (NTD; RDR2), S: 242/243- (NTD; RDR4), S:G1085A, N:M234I	Mycophenolate mofetil, tacrolimus	DM, Cirrhosis/NASH, CAD/HTN, HLD
P148	73/M	yes	yes	SOT-liver and kidney	BA.5.1	08/02/22	6	Convalescent plasma; dexamethasone and remdesivir for 5 days	/	A3078G	ORF1a:E938G (nsp3)	Mycophenolate mofetil, prednisolone, tacrolimus	AF/HTN, HLD
P14	70/M	yes	yes	CLL	AY.103	12/31/21	11	Casirivimab/imdevimab, dexamethasone and remdesivir for 5 days; 22 days prior	/	/	/	Ibrutinib	CAD, CKD, HLD
P16	78/M	yes	yes	CLL	BA.1	01/04/22	14	/	/	/	/	Acalabrutinib	DM, Heart failure/AF, HLD
P142	52/F	no	yes	Lymphoma	BA.5.2	07/28/22	1	/	/	/	/	Rituximab	HTN
P143	62/F	no	yes	SOT-kidney	BA.5.2.1	7/29/22	4	/	Nirmatrelvir/ritonavir, remdesivir for 5 days	/	/	Mycophenolate mofetil, prednisolone, tacrolimus	DM, heart attack/HTN, HLD
P155	77/F	no	yes	RA	BA.5.2	08/10/22	6	/	/	/	/	Etancercept	DM, heart attack/CAD/heart failure
P192	67/F	no	yes	RA	BA.5.2.1	11/04/22	13	/	/	/	/	Mycophenolate mofetil, prednisolone, tacrolimus	NAFLD, HTN, HLD, ILD
P71	83/F	no	no	/	BA.2	04/25/22	8	/	/	/	/	/	AF/HTN
P90	87/M	no	no	/	BA.2.12.1	05/23/22	1	/	Remdesivir for 3 days	/	/	/	DM, ERSD, CAD/heart failure/HTN, HLD, asthma
P83	33/M	no	no	/	BA.2.12.1	05/12/22	7	/	/	/	/	/	/
P125	77/M	yes	no	/	BA.5.2	07/13/22	0	Remdesivir for 5 days	Dexamethasone for 10 days, nirmatrelvir/ ritonavir 1 dose	/	/	/	DM, CKD, heart failure/CAD, HLD

*RA, Rheumatoid arthritis; SOT, Solid organ transplant; AML, Acute myeloid leukemia; CLL,Chronic lymphocytic leukemia;

**NAFLD, Nonalcoholic fatty liver disease; NASH, Nonalcoholic steatohepatitis; DM, Diabetes mellitus; ILD, Interstitial lung diseases; HLD, Hyperlipidemia; HTN, Hypertension; CKD, Chronic kidney disease; CAD, Coronary artery disease; ERSD, End-Stage Renal Disease; AF, Atrial fibrillation.
